# HEALTH TECHNOLOGY ASSESSMENT ON CERVICAL CANCER SCREENING, 2000–2014

**DOI:** 10.1017/S0266462315000197

**Published:** 2015

**Authors:** Betsy J. Lahue, Eva Baginska, Sophia S. Li, Monika Parisi

**Affiliations:** BD Health Economics and Outcomes Research; BD Health Economics and Outcomes Researchmonika_parisi@bd.com

**Keywords:** Technology assessment, Uterine cervical neoplasms, Mass screening, Vaginal smears, Diagnostic services

## Abstract

**Objectives:** The aim of this study was to conduct a review of health technology assessments (HTAs) in cervical cancer screening to highlight the most common metrics HTA agencies use to evaluate and recommend cervical cancer screening technologies.

**Methods:** The Center for Reviews and Dissemination (CRD), MedLine, and national HTA agency databases were searched using keywords (“cervical cancer screening” OR “cervical cancer” OR “cervical screening”) and “HTA” from January 2000 to October 2014. Non-English language reports without English summaries, non-HTA reports, HTAs unrelated to a screening intervention and HTAs without sufficient summaries available online were excluded. We used various National Institute for Health and Care Excellence (NICE) methods to extract key assessment criteria and to determine whether a change in screening practice was recommended.

**Results:** One hundred and ten unique HTA reports were identified; forty-four HTAs from seventeen countries met inclusion criteria. All reports evaluated technologies for use among women. Ten cervical screening technologies were identified either as an intervention or a comparator. The most common outcome metric evaluated was diagnostic accuracy, followed by economic effectiveness. Additional outcome metrics such as the use of adjunct testing, screening intervals, and age-specific testing were commonly evaluated. Nearly one-third (fifteen of forty-four) of HTAs recommended a change in practice.

**Conclusions:** This review highlights popular metrics used in HTAs for cervical cancer screening. Clinical and economic effectiveness metrics have been consistently assessed in HTAs, while the use of adjunct testing, screening intervals, and age-specific screening became increasingly prevalent from after 2007. Moreover, we observed an increase in optimized recommendations after 2007.

Cervical cancer is a major contributor to cancer-related morbidity and mortality worldwide. The World Health Organization estimates approximately 530,000 women develop cervical cancer, and 270,000 women die from the disease each year (1). Several epidemiological studies and heath technology assessments (HTAs) around the world have demonstrated the importance of regular cervical screening programs, which lead to early, effective, and cost-effective treatment of precancerous lesions (1). Organized screening programs are critical in identifying the disease before it enters advanced stages, when treatments are often less effective compared with early interventions (2–4). Such programs are an important public health and policy goal, as they have contributed to steadily declining mortality rates in many countries (1).

Population screening for cervical cancer was first introduced in the 1950s using a method called the Papanicolaou (Pap) smear (5), which is also known as conventional cytology. Since its widespread introduction, conventional cytology has been acknowledged for its role in decreasing the global incidence of cervical cancer and associated mortality around the world (6). Before the 1950s, cervical cancer was a leading cause of death in the United States among women of childbearing age. Between 1955 and 1992, the incidence of cervical cancer in the United States declined by nearly 60 percent (5). Despite the profound impact of conventional cytology on cervical cancer incidence, uncertainty around test sensitivity and the occurrence of false-positive diagnoses prompted ongoing development of new technologies. Now, screening women for cervical cancer can be accomplished using various technologies and methodologies including, but not limited to, visual inspection using acetic acid (VIA), conventional and liquid-based cytology (LBC), molecular tests involving HPV genotype detection, colposcopy, biomarker detection, electrical impedance probes, and computer-assisted systems. Table 1 includes a description of each technology included in this review.

**Table 1.** Definitions of Cervical Cancer Screening Technologies Included in This Review

Cervical Screening Technology	Methodology
Conventional cytology or Pap smear	A sample of cervical cells is taken using a spatula and transferred to a glass slide where they are sent to a laboratory for observation using a microscope. A cytotechnologist or physician will examine the cells for cervical abnormalities.^1^
Liquid-based cytology (LBC)	A sample of cervical cells is taken using a soft brush and transferred to a special preservation liquid. Similar to conventional cytology, the sample is sent to a laboratory for observation under a microscope, where the sample is examined for cervical abnormalities.^1^
Human papillomavirus (HPV) DNA testing	A molecular diagnostic test designed to detect the presence of high-risk HPV DNA.^2^
Human papillomavirus (HPV) mRNA testing	A molecular diagnostic test designed to detect the presence of HPV mRNA.^2^
Colposcopy	A colposcope, much like a microscope, is used for closer examination of the cervix. This procedure is typically performed after an abnormal cytology test or positive HPV test. The doctor may apply a dilute vinegar solution to the cervix causing the abnormal areas to turn white. A biopsy may also be performed.^2^
Colposcopic digital image analysis systems	Adjunctive technologies to colposcopy which use digital technology to help scan for cellular abnormalities.^3^
Biomarkers	An immune cytochemistry test to detect virally induced oncogenic molecular changes in the cell through staining of the certain biomarkers found to be overexpressed by HPV-infected cells.^4^
Epitheliometer	A probe technology which uses electrical impedance spectroscopy to detect cervical cancer and pre-cancerous changes of the cervix in real-time.^5^
Computer-assisted image analysis (CAIA)	A system designed to scan already prepared cervical cytology slides for abnormalities and has the ability to queue samples to point the cytologist to potential “low-risk” and “high-risk” slides.^6^
Visual inspection using acetic acid (VIA)	Acetic acid is applied to the cervix, where it is inspected with the naked eye for cervical abnormalities.^7^

^1^Mayo Clinic. Tests and Procedures: Pap Smear. http://www.mayoclinic.org/tests-procedures/pap-smear/basics/what-you-can-expect/prc-20013038.

^2^National Cancer Institute (NCI) Dictionary of Cancer Terms. http://www.cancer.gov/dictionary.

^3^ Wade R, Spackman E, Corbett M, Walker S, Light K, et al. Adjunctive colposcopy technologies for examination of the uterine cervix–DySIS, LuViva Advanced Cervical Scan and Niris Imaging System: a systematic review and economic evaluation. NIHR Health Technol Assess. 2013.

^4^ Kisser A, Zechmeister-Koss I. P16/Ki-67 dual stain in the triage of PAPIII/IIID cytology in cervical cancer screening. Vienna: Ludwig Boltzmann Institut fuer Health Technology Assessment (LBIHTA)., 2013; HTA-Projektbericht 72.

^5^ Adelaide Health Technology Assessment (AHTA). Zilico hand-held device for rapid cervical cancer detection. Australia and New Zealand Horizon Scanning Network (ANZHSN). 2010.

^6^ Willis BH, Barton P, Pearmain P, Bryan S, Hyde C. Cervical screening programmes: can automation help? Evidence from systematic reviews, an economic analysis and a simulation modelling exercise applied to the UK. Health Technology Assessment. 2005; 9(13).

^7^ University of Zimbabwe/JHPIEGO Cervical Cancer Project. Visual inspection with acetic acid for cervical-cancer screening: test qualities in a primary-care setting. Lancet. 1999; 353: 9156.

Unlike other cancers, cervical cancer is the first cancer known to be virally induced, as the majority of diagnoses are associated with a select few types of human papillomavirus (HPV) genotypes (7). In addition to organized screening programs, many countries have implemented primary prevention initiatives including HPV vaccination (8).

As new tests, systems, and algorithms are developed for managing cervical cancer screening programs, policy makers have a responsibility to monitor the body of robust evidence and apply changes to these programs when warranted. It is essential that government endorsed algorithms are both supported by evidence and accepted by the community of women that the programs are intended to serve. In many countries, HTAs are designed to bring evidence to bear on public health policy funding and implementation, informing the decision-making process that surrounds the adoption of new technologies and new clinical algorithms around screening or vaccination. Several national and regional HTA bodies have completed systematic evidence reviews and developed decision models to inform policy makers in implementing cervical screening guidelines.

The aim of this study was to identify all published HTAs in cervical cancer screening from 2000 to 2014 and to report the most common metrics used by HTA agencies when evaluating a new technology in this space. We abstracted information on the population of women included in the reviews, the interventions and comparators that were examined, and how various clinical and economic effectiveness outcomes were measured. Moreover, we indicated if a recommended change in practice resulted. Based on the results of our review, we highlight some of the emerging trends regarding this public health initiative, as well as some of the challenges HTA bodies face during this resource intensive process.

Our study is the first of its kind to review HTAs in cervical cancer screening and to centralize key components of all such reports in one article. Our review can inform users of HTAs, such as policy makers and manufacturers, of the most common metrics used across geographies to assess new cervical cancer screening technologies. Furthermore, our study can inform HTA agencies responsible for carrying out these assessments of some of the current trends in cervical cancer HTA.

## METHODS

### Data Sources

We conducted a review of HTA reports published between January 1, 2000, and October 31, 2014. The search was conducted using the following databases: Center for Reviews and Dissemination- University of York (CRD) and MedLine. Additionally, the following country agency Web sites were searched: USA (AHRQ and USPSTF), Canada (CADTH), France (ANAES), Australia (MSAC), United Kingdom (NICE and NHS/NIHR), Sweden (SBU), Belgium (KCE), Denmark (DACEHTA), Germany (IQWiG/DAHTA-DIMDI), New Zealand (NZHTA). The search terms used were (“cervical cancer screening” or “cervical cancer” or “cervical screening”) AND “Health Technology Assessment.” The search strategy and information sources are detailed in Supplementary Table 1.

### Study Selection Criteria

Identified HTA reports were initially screened to determine whether the primary focus of the report was related to cervical cancer screening. HTAs focused on vaccination were not included in this review. HTAs were also excluded if they were in a language other than English without a substantial English summary, not an official HTA report, or did not have a full report publically available.

### Data Extraction

We abstracted key parameters from each HTA report based on the PICO process employed by the National Institute for Health and Care Excellence (NICE):

(P)opulation: Who is the population for which the technology is intended?
(I)ntervention: What is the main intervention being evaluated?
(C)omparators: What are the comparators used in the report?
(O)utcomes: What types of outcomes were measured?

We aimed to understand what metrics are most frequently used to evaluate the role of new technologies within a cervical cancer program, and to summarize trends in recommendations based on our interpretation of the data. In determining whether a change in screening practice was recommended, we used the NICE technology appraisal recommendation categories for guidance around how to classify the findings of each HTA and interpreted the conclusions of all included HTAs to the most appropriate category. The four NICE technology appraisal categories are as follows:

*Recommend*: The technology is recommended for use: (i) In line with the marketing authorization from the European Medicines Agency (EMA) or Medicines and Healthcare Products Regulatory Agency (MHRA) or (ii) In line with how it is used in clinical practice in the NHS (or both).

*Optimized*: The recommendations have a material effect on the use of a technology, and it is recommended for a smaller subset of patients than originally stated by the marketing authorization.

*In research only*: The technology is recommended for use only in the context of a research study, for example a clinical trial.

*Not recommended:* The technology is not recommended. In most instances, a technology will not be recommended if there is a lack of evidence for its clinical effectiveness or if the technology is not considered to be a cost-effective use of NHS resources, compared with current NHS practice.

We conclude that a change in practice was endorsed if the report's conclusion fell into the first or second category above (“Recommend” or “Optimized”). These two categories are not mutually exclusive, as some HTAs assessed multiple technologies and made broad recommendations for the majority of the population, with an optimized recommendation for a small subset of the population.

## RESULTS

**Figure 1.**
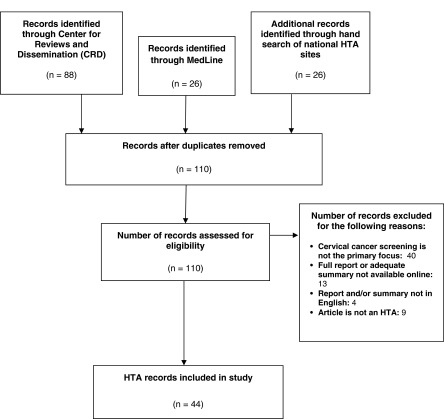
HTA Report Selection. HTA reports were identified through the Center for Research and Dissemination (CRD), MedLine, and country-specific HTA databases. All searches were limited to years 2000–2014.

The search identified 110 unique HTA reports (see Figure 1). Forty reports were excluded because the primary focus did not pertain to cervical cancer screening and nine reports were excluded because they were a form of peer-reviewed literature rather than a formal HTA report. Thirteen reports were eliminated because they were not accessible or did not offer an adequate summary online. Finally, four reports were excluded because the report and/or a sufficient summary were not available in English.

Forty-four discrete, full-text or summarized HTA reports with a principal focus on cervical cancer screening met full study criteria and were included for analysis. The included reports came from seventeen countries: United Kingdom (eight); Australia (eight); Germany (five); Spain, Italy, and Canada (three each); Sweden, New Zealand, United States (two each); and Austria, South Korea, Thailand, France, Belgium, Netherlands, Scotland, Denmark (one each).

### (P)opulation

All reports carried out their assessment on the female population, only. The majority of reports did not specify an age range for screening, but rather described the intended population as women of screening age. For those that did specify an age range, the range was from 14 years old to 70 years old across all reports. The vast majority of HTAs included in this assessment evaluated new technology for a primary screening population, while six reports evaluated new technology for use as a triage tool for more targeted screening after an initial diagnosis had been made.

### (I)ntervention and (C)omparators

Ten cervical screening interventions were evaluated across all forty-four reports: two cytology tests (conventional cytology and LBC), two HPV molecular tests (DNA and mRNA), colposcope, adjunctive colposcopic digital image analysis systems, oncogenic biomarker detection and staining, electrical impedance device, computer-assisted image analysis (CAIA), and visual inspection using acetic acid (VIA).

Of the ten cervical screening technologies evaluated across these forty-four reports, the most common intervention of interest from 2000 to 2014 was the HPV DNA test, with eleven reports evaluating this technology as a primary or triage test. Nine reports evaluated LBC as a primary screening intervention, while eight reports evaluated the introduction of CAIA for use as a primary screening intervention. Rather than evaluating the implementation of one intervention compared with the standard of care, ten reports evaluated a combination of cytology and HPV testing to assess the optimal placement of each technology in a population screening algorithm.

**Figure 2.**
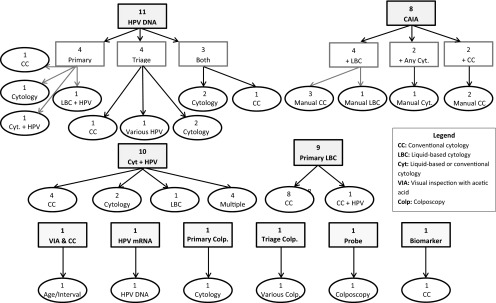
Interventions and their respective comparators from 44 included HTAs. The interventions and their sub-categories are in rectangles, while the comparators are in ovals.

In addition to cytology and HPV DNA testing, there were a few reports that evaluated less common screening interventions. One report from Spain in 2010 compared the use of mRNA testing with that of HPV DNA testing (9). One report from Germany in 2007 evaluated the use of colposcopes in a primary screening population (10), and another report from the United Kingdom in 2013 compared the use of various methods and technologies that work in conjunction with a colposcope (11). Another report from Austria in 2013 evaluated a new biomarker technology for use as a triage for abnormal cytology (12). Moreover, a 2010 HTA from Australia evaluated the use of an electric impedance probe as an adjunct to primary colposcopy screening (13). Finally, one report from Thailand in 2008 evaluated primary screening with the use of VIA and conventional cytology at various ages and screening intervals (14). All interventions and comparators can be found in Figure 2.

### (O)utcomes

#### Clinical Effectiveness

Forty-three of the forty-four HTAs evaluated diagnostic accuracy using test sensitivity and specificity as the primary measure of clinical effectiveness. One HTA did not specifically assess diagnostic accuracy because the focus of the report was on productivity related to CAIA (15). Of the reports that did assess the diagnostic accuracy of a technology, an evaluation was made between the main intervention(s) and comparator(s).

Twenty-nine reports compared sensitivity and specificity to histological outcomes (8;11–14;16–36), 76 percent (twenty-two of twenty-nine) of which assessed cervical intraepithelial neoplasia CIN2+ or CIN2/3+ as the endpoint of interest (8;9;11–13;16–18;23;25–27;29–34;36;37). Six reports analyzed sensitivity and specificity to cytological outcomes such as low grade squamous intraepithelial lesions (LSIL) or high grade squamous intraepithelial lesions (HSIL) (22;23;36;38–40). There were three reports that compared both histological and cytological outcomes (22;23;36).

In addition to diagnostic accuracy, eight reports also examined cervical cancer incidence (8;14;20;26;28;37;39;41), and five reports examined mortality due to cervical cancer as clinical endpoints (8;26;28;37;41). Finally, specimen quality was examined in 45 percent (twenty of forty-four) of all reports (8;14;19;20;22–26;30–32;39;40;42–47).

#### Economic Effectiveness

**Figure 3.**
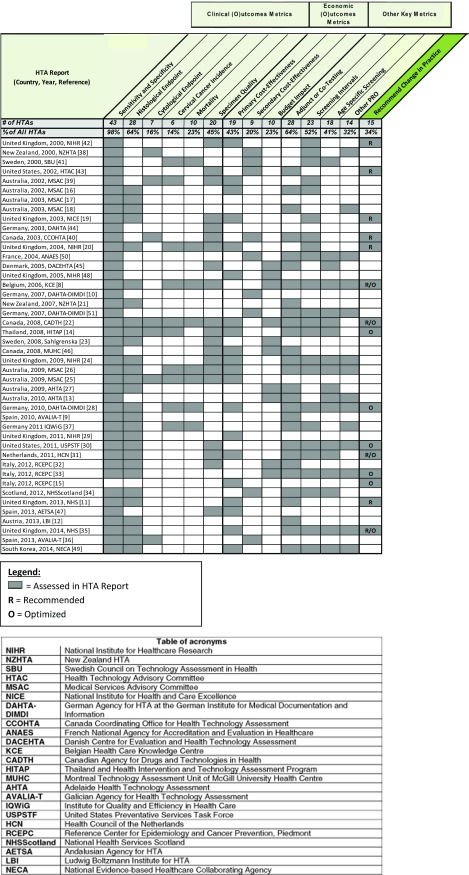
Common metrics assessed by each cervical cancer HTA and whether a recommended change in practice was made.

In total, thrity-six of forty-four HTAs carried out a health economic analysis. A primary cost-effectiveness analysis was the most commonly used health economic model, with twenty HTAs carrying out this type of analysis (11;14–16;9;20;22;24–26;28;29;31;35;39;42;46–49). Nine HTAs conducted a secondary cost-effectiveness analysis (10;34;36;38;40;41;43;50;51), seven HTAs developed a budget impact model (8;13;23;27;32;33;45), and three HTAs carried out both a cost-effectiveness and budget impact analysis (22;46;48). A major factor that contributed to the economic effectiveness of a new technology was related to the increased productivity it would bring to the laboratory, a metric which was evaluated in nine reports (15;19;20;25;29;32;36;42;43). Highlighted economic metrics are summarized in Figure 3.

#### Other Key Outcomes Metrics

As new technologies have emerged over the years, the complexity of evaluating new interventions grew in scope. Aside from clinical and economic outcomes, common metrics we extracted from the forty-four reports include the evaluation of certain technologies as an adjunct to other tests, screening interval impact due to new technology, age specific interventions, and the impact on patient reported outcomes.

In total, there were twenty-eight reports which examined adjunct testing for use with the main intervention, either as a triage or co-test (8;9;11–14;16;18;22;24;26–28;30–35;37;40;43;45;46;49–51). Of the forty-four HTA reports included in this review, 50 percent (twenty-two of forty-four) examined modifying testing intervals for various screening technologies to find optimal screening algorithms (8;14;16;19;20;22;24;26;28;30;31;33–35;37–42;49–51). Furthermore, rather than assessing one screening program for all women of screening age, seventeen HTAs considered a patient's age group in making specific screening recommendations (8;13;14;22;24;26–28;30;31;33;34;41;45;49–51). Twelve reports also considered the impact of introducing a new test on a patient's stress and anxiety through Patient Reported Outcomes (8;13;18;24;26;28;33;35;37;38;50;51), a common metric used in HTAs evaluating HPV tests. A summary of these important outcome metrics can be found in Table 2.

### Was a Change in Screening Practice Recommended?

Based on our analysis of the forty-four included reports, fifteen HTAs from five countries recommended a change in practice for their cervical cancer screening program. It is important to note that not all HTAs seek to formally make recommendations, and some exist to simply summarize all existing evidence in data. Four reports had both a recommendation for a broader population and a subset of the population, and thus fell into the categories of “Recommend” and “Optimized.” Six reports were “Recommend” only, and five reports were “Optimized” only.

“In research only” was a finding of one report from the year 2000, which concluded that HPV testing should only be used within the framework of clinical trials. Finally, twenty-nine HTA reports fell into the “Not Recommend” category. Reasons for this conclusion could include insufficient evidence, poor clinical/economic effectiveness, or that the objective of the HTA was to simply summarize data rather than formally recommend a technology and/or change in practice. Figure 3 details the findings of each included HTA.

## DISCUSSION

This review is the first of its kind in the area of cervical cancer HTAs, and provides insights into some of the most common metrics used to evaluate new cervical cancer screening interventions under consideration by national and regional HTA bodies. Our review found that the patient age range evaluated in cervical cancer screening HTAs did not change over time. Conversely, the complexity of evaluating new interventions against their comparators expanded in scope as new technologies emerged in this space. We found that HTAs published in the earlier years of this review typically focused on the introduction of one new technology and how it compared with the standard of care; however many of the more recently published HTAs sought to evaluate the optimal placement of various technologies in more complex cervical cancer screening algorithms.

In terms of outcome metrics, diagnostic accuracy assessed by sensitivity and specificity was the most consistent outcome evaluated in cervical cancer HTAs, as only one HTA did not address it. Moreover, our review found that cost-effectiveness was also an important metric, as it was assessed in 68 percent of included HTAs. This is due in large part to the wide population which screening technologies are intended to serve. Notably, of the fifteen HTAs that recommended a change in practice, twelve carried out a cost-effectiveness analysis, of which 100 percent yielded favorable cost-effectiveness results, highlighting the importance of this metric in the assessment process.

Other commonly assessed outcome metrics include the use of adjunctive technologies, the adjustment of screening intervals, and age-specific screening algorithms. We recognized an increasing trend toward the inclusion of these three outcome metrics over time. Before 2007, only 13 percent of HTA reports evaluated these three factors; yet from 2007 to 2014, 43 percent of HTAs considered all three. It is important to highlight the significance of 2007, as this was when a new prevention technology, the HPV vaccine, was brought to market, which likely encouraged HTAs to consider multiple factors in finding an efficient approach for cervical cancer prevention in their populations.

As new technologies have become available for screening, algorithms around how to deploy them to an entire screening population have become increasingly complex. From 2000 to 2014, we saw an increase in optimized recommendations. Specifically, nearly 90 percent (eight of nine) of recommendations made after 2007 involved an optimized component to the recommendation, with seven of eight recommending more than one technology type for use (Figure 3). With the breadth of available technologies that have emerged over the last decade to address this preventable disease, HTAs appear to recognize that there is not a “one-size-fits-all” approach to prevention.

The process of conducting an HTA is a resource-intensive undertaking. For those who are responsible for carrying out HTAs, standardization and information sharing is endorsed by global HTA groups and several cooperative agreements (52;53). Information sharing, specifically findings of a literature review, could serve as a resource to other HTA bodies looking to conduct an assessment of a given technology. This could reduce the time and initial resources required to conduct an HTA, potentially allowing for more time to focus on system-specific funding and implementation needs. Our review can serve as a reference tool of published cervical cancer HTAs from 2000 to 2014 so that agencies that conduct HTAs can be made aware of previous assessments carried out in this disease area. Moreover, our study helps to highlight some of the current trends in cervical cancer HTA.

## LIMITATIONS IN THE STUDY

Several limitations were identified in this study. Given that only studies available in English were included in this review, some valuable HTA reports in other languages may have been overlooked, which may have biased our selection process. However, we only identified four HTAs that were eliminated due to language so this would have minimal bias to the trends we identified. Second, this literature review is on HTA reports, which may contain conclusions that are not peer-reviewed. As such, the forty-four HTA reports varied in the quality of evidence considered. Gathering supporting evidence presents a challenge in this disease area, as large sample sizes and long time frames are required, technology is continually evolving, and blinding is virtually impossible. Additionally, generalizations were made regarding the various screening technologies because most reports did not specify the test brand. This could potentially discount the differences in specific technology characteristics. Notably, this review excluded an important development in cervical cancer prevention. HPV vaccination is not a cervical cancer screening technology, and therefore was not included in this study. This limitation did not have a substantial impact because little data on HPV vaccination was available to HTAs in our time period. Finally, our review does not report on whether a new policy was implemented due to findings of the included HTAs.

### Conclusion

From 2000 to 2014, we found forty-four HTAs from seventeen countries that evaluated the role of cervical cancer technologies in population screening. Based on this review, assessing available technologies based on clinical and economic effectiveness have been consistent metrics over the last fourteen years. Examining other factors, such as adjunct testing, screening intervals, and patient screening age became increasingly prevalent in the decision-making process between 2000 and 2014, as we also observed an increase in optimized recommendations. HTA processes that incorporate multiple technologies and screening algorithms provide a holistic approach to impact policy change. Moreover, there is an opportunity for HTA agencies to look to neighboring countries to accelerate learning in this rapidly evolving field. While it is critical for economic analyses to be locally relevant, there may be opportunity to harmonize and reduce variability in clinical effectiveness reviews. Finally, there is a need for future HTAs to include robust analyses that examine the impact of HPV vaccinations.

## POLICY IMPLICATIONS

Manufacturers and policy makers are often the end users of HTA reports, as HTA recommendations can translate to positive funding and access to technologies with the implementation of new guidelines. This is the first global assessment of HTA reports for cervical cancer screening technologies. We identified key metrics used when evaluating new screening technologies within a cervical cancer screening program, and highlighted evolving metrics used across geographies. It is important for manufacturers to understand the criteria used by HTAs to evaluate new technologies, as this can inform research and development (R&D) of the metrics and outcomes expected when bringing a new innovation to market. Additionally, it is important for national and regional policy makers to efficiently assess opportunities to reduce the burden of cervical cancer by regularly monitoring new evidence and understand how the evidence was assessed. By understanding this global body of evidence, evaluating common metrics, and reporting effective program elements, manufacturers can develop innovative solutions that will best serve their patient populations, and policy makers can better guide best practices to reduce the burden of cervical cancer within their populations.

## Supplementary material

For supplementary material accompanying this paper visit http://dx.doi.org/10.1017/S0266462315000197. click here to view supplementary material

## CONFLICTS OF INTEREST

The authors of this research are employed by Becton, Dickinson and Company, a manufacturer of diagnostic and imaging technologies used for cervical cancer screening.
