# Supplementary material for: HEALTH TECHNOLOGY ASSESSMENT ON CERVICAL CANCER SCREENING, 2000–2014
Source: Int J Technol Assess Health Care. 2015;31(3):171–80. doi: 10.1017/S0266462315000197 (PMC4535324; doi:10.1017/S0266462315000197)
Supplement: Supplementary file 1 [file S0266462315000197sup001.docx]

**Supplementary Table 1: Systematic Review Search Parameters.** We searched CRD (University of York), MedLine and national HTA agency websites for HTAs published from January 2000 – October 2014

Total = 44 HTAs from 11 countries

| **Search Database** | **Search Parameters** | **Results** |
| --- | --- | --- |
| CRD Database | Search Term: “cervical cancer screening” OR “cervical screening” OR “cervical cancer”  Restrict to Database: HTA  Publication Year: 2000 – 2014  Record Types: Published HTAs | **Hits:** 88  **Met Inclusion Criteria:** 37  **Removed as duplicate:** 1  **Included from this search:** 36  **Excluded:**   - **Not an HTA:** 1 - **Cervical cancer screening not primary focus:** 33 - **Summary not publically available:** 13 - **Summary not available in English:** 4 |
| MedLine | Search Term: "Health technology assessment" AND ("cervical cancer screening" OR "cervical screening" OR "cervical cancer")  Publication Year: 2000 – 2014 | **Hits:** 26  **Met Inclusion Criteria:** 12  **Removed as duplicate:** 8  **Included from this search:** 4  **Excluded:**   - **Not an HTA:** 7 - **Cervical cancer screening not primary focus:** 7 |
| National HTA agency websites | “cervical cancer screening” – some sites required navigating to search box, others did not allow search terms so a hand search was conducted  Publication year 2000-2014  USA (AHRQ and USPSTF), Canada (CADTH), France (ANAES), Australia (MSAC), U.K. (NICE and NHS/NIHR), Sweden (SBU), Belgium (KCE), Denmark (DACEHTA), Germany (IQWiG/DAHTA-DIMDI), New Zealand (NZHTA) | **Met Inclusion Criteria:** 26  **Removed as duplicate:** 21  **Included from this search:** 4 |
